# Concepts Collide: Genomic, Immune, and Microbial Influences on the Tumor Microenvironment and Response to Cancer Therapy

**DOI:** 10.3389/fimmu.2018.00946

**Published:** 2018-05-04

**Authors:** Miles C. Andrews, Alexandre Reuben, Vancheswaran Gopalakrishnan, Jennifer A. Wargo

**Affiliations:** ^1^Department of Surgical Oncology, The University of Texas MD Anderson Cancer Center, Houston, TX, United States; ^2^Olivia Newton-John Cancer Research Institute, Heidelberg, VIC, Australia; ^3^School of Cancer Medicine, La Trobe University, Heidelberg, VIC, Australia; ^4^Department of Genomic Medicine, The University of Texas MD Anderson Cancer Center, Houston, TX, United States

**Keywords:** cancer immunotherapy, biomarkers, heterogeneity, microbiome, cancer genomics, tumor microenvironment, systemic immunity

## Abstract

Cancer research has seen unprecedented advances over the past several years, with tremendous insights gained into mechanisms of response and resistance to cancer therapy. Central to this has been our understanding of crosstalk between the tumor and the microenvironment, with the recognition that complex interactions exist between tumor cells, stromal cells, overall host immunity, and the environment surrounding the host. This is perhaps best exemplified in cancer immunotherapy, where numerous studies across cancer types have illuminated our understanding of the genomic and immune factors that shape responses to therapy. In addition to their individual contributions, it is now clear that there is a complex interplay between genomic/epigenomic alterations and tumor immune responses that impact cellular plasticity and therapeutic responses. In addition to this, it is also now apparent that significant heterogeneity exists within tumors–both at the level of genomic mutations as well as tumor immune responses–thus contributing to heterogeneous clinical responses. Beyond the tumor microenvironment, overall host immunity plays a major role in mediating clinical responses. The gut microbiome plays a central role, with recent evidence revealing that the gut microbiome influences the overall immune set-point, through diverse effects on local and systemic inflammatory processes. Indeed, quantifiable differences in the gut microbiome have been associated with disease and treatment outcomes in patients and pre-clinical models, though precise mechanisms of microbiome-immune interactions are yet to be elucidated. Complexities are discussed herein, with a discussion of each of these variables as they relate to treatment response.

## Introduction

Interest in defining factors that influence the outcome of cancer therapy has existed for as long as the therapies themselves. Traditionally, a highly tumor-centric focus has dominated, resulting in a now well-characterized yet still incomplete view of the complex molecular and cellular tumoral dynamics relevant to cancer progression and to treatment response.

Several of these factors, particularly the overall somatic mutational burden of the tumor, have gained traction and even potential clinical utility in the prediction of response to immunotherapy, notwithstanding ongoing concerns about their limited accuracy. Qualitative genomic characterization of tumors may also be very informative, however, information derived from such analyses is subject to the limitations imposed by sampling error and heterogeneous composition of synchronous tumors in patients with multiple metastases. Despite initial enthusiasm, an appreciation of the limitations of genomic characterization alone is emerging, and a more comprehensive analysis of the multitude of factors influencing therapeutic responses is critically needed.

In this mini review, we provide an overview of genomic factors implicated in the response to cancer immunotherapy, utilizing melanoma as a model “immunogenic” tumor from which the majority of empirical evidence derives. We will also discuss immune determinants of response and resistance, highlighting recent data regarding tumor immune cell co-evolution influenced by immunogenic factors arising from tumor cells, and the immunoediting effects of the responding immune infiltrate, both of which are impacted by intra- and inter-tumoral heterogeneity. In addition to this, we will complement studies of the tumor microenvironment to better delineate the crosstalk between the tumor microenvironment and overall host immunity with the microbiome, as this has been shown to influence outcomes ranging from tumor growth and immunity to treatment-related response and toxicity. Though discussion of the gut microbiome will predominate, we will also describe the potential impact of the intra-tumoral microbiome on resistance to cancer therapy, thus providing a full discussion of the intersection of tumor genomics, immunity, and the microbiome in shaping therapeutic responses, as summarized in (Table 1).

**Table 1:** Inter-relationships between clinical, genomic, immune, and microbial factors drawn from the patient (systemic), tumor microenvironment (histology), and disease-level domains, with associated influence on immunotherapeutic outcomes.

	Clinical	Genomic	Immune	Microbial	Therapeutic
**Patient/systemic**	**Age**	*Accumulated mutations*	Immune senescence		May impact treatment decisions
	
	**Comorbidities**		Iatrogenic immunosuppression (e.g., steroid use)	Iatrogenic dysbiosis (e.g., antibiotic use)	May limit treatment options and drug interactions
	
	**Performance status**				May limit treatment options
	
	**Environmental exposures**	Carcinogen exposures (e.g., UV and tobacco smoke) → DNA damage, accumulation of mutations		Microbe-derived genotoxins (e.g., pks/colibactin)	
	
		Th1/Th17 vs Th2 skewing and effects on anti-cancer immunosurveillance	**Promotion of Th1/Th17 responses by gut microbiota** (e.g., *via* DAMPs/PAMPs)	Potentially oncogenic but also permissive to immunotherapy response
	
	Diet/stress/antibiotic use		*Immunosuppression*	**Dysbiosis**	
	
			Permissive effect on anti-cancer T cell function	**Myeloid priming**	
			
				**Gut microbial diversity** (alpha diversity)	Associated with immunotherapy response
			
			Regulation of immune tone, FoxP3+ Treg maintenance	**Microbial metabolites** (e.g., short-chain fatty acids)	

**Tumor microenvironment/histology**	**Cancer type and sub-type**	Mutational load, specific mutations, mutational and multi-“omic” signatures (e.g., carcinogen-related)	Affects intrinsic immunogenicity	Influenced by exposure to local microflora	Expectation of immunotherapy outcome markedly influenced by cancer histology and sub-type (e.g., mucosal vs cutaneous melanomas)
	
	**Immunohistochemical PD-L1 scoring** (note: variable antibody performance and individualized thresholds for clinical interpretation)	Cancer-associated molecular pathways influence immunoregulatory molecule expression	**PD-L1** status, expression of additional checkpoint receptors/ligands on T cells and tumor		Positive predictive value for PD-(L)1 inhibitor based therapy
	
	Immunohistochemical evaluation		**Lymphocytic infiltration**	Enrichment of specific taxa in gut microbiome associated with CD8+ TIL	Presence of TIL associated with better prognosis across many cancer types
	
			**Presence of immunoregulatory or suppressive cell subsets** (e.g., Treg, MDSC, and TAF/TAM)	Enrichment of specific taxa in gut microbiome associated with suppressive cell populations in the tumor	Poor immunotherapy response unless specifically targeted by the immunotherapeutic agent
		
		**HLA types and diversity**	Formation of immune synapses, neoantigen presentation, need to optimally match T cell repertoire		HLA diversity associated with improved survival following checkpoint blockade therapy
		
		**HLA class I loss**	Immune evasion		
		
		Altered antigen presentation machinery, EMT-like plasticity (e.g., IFN-driven proteasomal alteration)	**Inflamed microenvironment**	Influenced by gut microbial composition and local/intra-tumoral microflora	Differential effects on anti-cancer immunity depending on time course (e.g., acute vs chronic/persistent inflammation)

		**Defective antigen presentation machinery** (e.g., *JAK2* mutations and β*2M* loss)	Loss of antigen presentation, immune evasion		
		
		Adaptive mutational/neoantigen pruning and immunoediting	**T cell repertoire** (e.g., clonality, neoantigen-specific clones)		
		
		Altered transcriptome and/or methylation patterns		**Locally pro-inflammatory microbes**	
		
				**Intra-tumoral microbial metabolism**	*In situ* degradation of chemotherapeutic agents

**Disease**	**Stage**	Mutational load, specific mutations, and mutational signature (e.g., carcinogen-related)	Progression-related antigenic change, clonal selection (e.g., under influence of spontaneous anti-cancer immunity or prior therapy)	*Tumor-induced dysbiosis*	
	
	**Burden of disease**	*Underlying inter-tumoral genomic heterogeneity*		*Tumor-induced dysbiosis*	May influence fitness for treatment, adversely prognostic
	
	Growth characteristics (e.g., rate of progression and metastatic site tropism)	**Driver mutation status** (e.g., *BRAF^V600^*), specific **methylation and copy-number alterations**	Immune pathway modulation (e.g., by MAPK activation), tumor antigen expression (e.g., modulated by EMT-like processes)	Methylation and transcriptome alterations associated with (local) microflora	Aggressive disease, certain sites of involvement (e.g., brain) adversely prognostic
	
	Associated with some clinical characteristics (e.g., carcinogen type- and dose-related and lower overall mutational burden in presence of clear driver mutations like *BRAF^V600^*)	**Total mutational burden**	Neoantigen repertoire		Predictive of response to checkpoint blockade (monotherapy), unclear relationship for combinations at this stage
	
		**EMT-like plasticity**	Evolution of potential tumor antigen expression (e.g., melanoma differentiation antigens and cancer-testis antigens)	*Microbial effects on methylation known, potential for dynamic epigenomic influences*	Drug sensitivity, immune vulnerability
		
		**Immune exclusion** (e.g., β-catenin)	Failure of effector immune cell infiltration, “immune-desert”		

*Core concepts are shown in bold. Entries in italics represent speculative interactions*.

*TAF, tumor-associated fibroblasts; TAM, tumor-associated macrophages; TIL, tumor-infiltrating lymphocytes; Treg, regulatory T cells; DAMP, damage-associated molecular patterns; EMT, epithelial-to-mesenchymal transition; MDSC, myeloid-derived suppressor cells; PAMP, pathogen-associated molecular pattern*.

## Tumor-Specific Influences on Response

**Figure 1:**
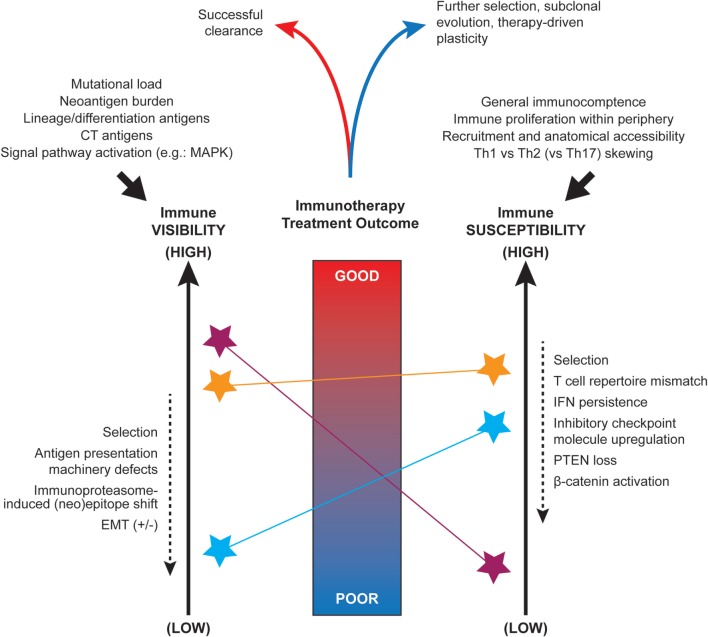
Factors influencing the immune visibility and susceptibility of tumors. Nomogram-conceptualization of the competing influences of tumor immune visibility (*at left*) and the susceptibility of tumor cells to immune attack (*at right*). Due to underlying intra- and inter-tumoral heterogeneity, distinct tumor cell sub-clones or microenvironments (*denoted by colored stars*) may display a range of visibility and susceptibility characteristics that must be integrated when predicting the overall outcome of spontaneous or immunotherapy treatment-induced anti-tumor responses. The initial set-point of immunogenicity is influenced by several factors including somatic mutations, antigen expression, and signal pathway activity (*top left*). The anti-tumor immune set-point is similarly influenced by a number of systemic factors such as availability of immune cell populations for recruitment and Th1 skewing (*top right*). Multiple factors have been implicated as dynamic modulators of these visibility and susceptibility states (*dashed arrows at sides*).

Long before the advent of modern genomic technologies, histologic sub-types of cancer were described, with differences in response to therapeutic intervention noted across these sub-types. This is well illustrated in melanoma, for which several clinicopathologic sub-types exist, including superficial spreading, acral, desmoplastic, and mucosal melanomas. With the advent of next generation sequencing, we have gained tremendous insight into the molecular underpinnings of these clinicopathologic observations and into the mechanisms driving differences between tumors themselves. Distinct genomic aberrations frequently define histologic sub-types and can confer notable differences in therapeutic sensitivity that have major clinical relevance (1, 2). Beyond this, other components of the tumor microenvironment have been noted to play a major role in therapeutic response and resistance, impacting upon tumor visibility and susceptibility (Figure 1), as discussed below.

## Tumor Immune “Visibility”

In addition to their influence on oncogenic signaling and proliferative potential, genomic mutations present in melanoma and other cancers may have a profound impact on anti-tumor immunity and can contribute to the “visibility” of a tumor to the immune system (3, 4). This is largely shaped by the antigenic characteristics of the tumor cells allowing their recognition by the immune system, but may be shaped by other influences of these oncogenic mutations on the tumor cells themselves as well as the microenvironment. This is important, as therapeutic targeting of oncogenically activated signaling pathways may alter anti-tumor immunity. A key example of how genomic alterations may impact tumor visibility is illustrated in the case of *BRAF*-mutant melanoma. Early observations demonstrated a link between MAPK signaling and the expression of melanoma-associated antigens (5, 6), with subsequent data revealing brisk infiltration of tumors with T lymphocytes in the setting of treatment with BRAF inhibitor-based therapy (3, 7). Interestingly, inhibition of oncoproteins such as BRAF may also be associated with increased expression of HLA molecules and heat shock proteins, which can further contribute to a tumor’s visibility (8, 9).

More generally, tumor cell immune visibility is fundamentally dependent on the presence (or absence) of molecular moieties that can be recognized by components of the host immune system. Tumor cell self-antigens represent the basis of cognate interactions with cellular elements of the adaptive immune system, but have varying degrees of tumor cell specificity. Such antigens include differentiation or lineage-specific antigens, aberrantly expressed antigens either absent or found at only low levels in adult tissues (3), or may be truly tumor cell-specific neoantigens derived from the protein products of somatically mutated genes. Tumor neoantigens are felt to predominantly mediate effective anti-tumor immune responses because neoantigen-reactive T cells escape deletion mechanisms during T cell ontogeny, and respond to these antigens as “foreign” rather than “self” (10, 11). In addition, epithelial-to-mesenchymal-like (EMT-like) plasticity in melanoma is thought to contribute to functional and antigenic variation that has the potential to influence the efficacy of immune-based therapies (12). Given the prominent role of the lymphocyte response to MAPK blockade in *BRAF*-mutated melanoma, these EMT-like shifts in melanoma cell state may well also contribute to BRAF inhibitor resistance at least in part by altering melanoma cell visibility *via* this antigenic shift (13).

Tumor genomics gains specific relevance to immune visibility in light of the significance of tumor-specific neoantigens in shaping immune responses. The mutational landscape varies across tumor types (14), and is shaped by factors influencing carcinogenesis such as UV irradiation and smoking (14). Interestingly, responses to immunotherapy are positively associated with the mutational burden of each particular tumor type, evidenced by higher response rates and clinical benefit in tumor types with an overall high mutational burden, such as melanoma, non-small cell lung cancer (NSCLC), clear cell renal cell cancer, and genitourinary cancers (14, 15). A recent study of 151 patients with predominantly melanoma (34%) or NSCLC (24%), mostly treated with anti-CTLA-4, anti-PD-1, or anti-PD-L1 blockade therapy, assessed the relationship between tumor mutational burden measured by hybrid capture next generation sequencing and clinical outcomes. Using pre-defined cut-offs, patients with higher tumor mutational burden experienced higher response rates and longer progression-free and overall survival than those with low to intermediate tumor mutational burden (16). These results were broadly applicable to the sub-group of patients (42% of the overall cohort) with non-melanoma/non-NSCLC histologies. Interestingly, the relationship between higher mutational load and better treatment outcomes was not evident for patients who received combined anti-CTLA-4 and anti-PD-1 therapy. Further supporting the tumor mutational burden-response relationship are tumors with microsatellite instability and mismatch-repair deficiency, which demonstrate an increased sensitivity to checkpoint blockade likely to be related to an associated increase in mutational load and neoantigen burden (17, 18). Indeed, demonstration of microsatellite instability-high or mismatch-repair deficient tumors upon biomarker testing forms the basis for the first site-agnostic drug approval made by the FDA, for anti-PD-1 therapy. The practical limitations of measuring tumor mutational burden for use as a predictive biomarker before therapy have been significantly met by robust estimation of overall mutational load using data obtained from targeted next generation sequencing technologies that are now relatively widely accessible in the clinic (19, 20). In addition, cancer-gene panel mutational profiling by liquid biopsy represents a promising alternative mutational burden-related methodology for predicting immunotherapy response, as reported in an analysis of NSCLC patients enrolled in clinical trials of the anti-PD-L1 agent atezolizumab (21).

However, the relevance of a tumor cell’s mutational repertoire to immune dynamics is moderated by additional factors that affect expression, processing, and intrinsic immunogenicity of any putative neoantigen. The complex processes involved in cleaving a peptide, loading it onto an MHC molecule, transporting it to the cell surface, and ensuring its stability are essential to induce the antigenic T cell responses required for tumor clearance. Epitope production is influenced by the molecular chaperones and proteasomal machinery involved in protein processing; not all epitopes produced may be immunogenic, and a form of stochastic competition between display of immunogenic and non-immunogenic epitopes may ensue. The initially beneficial IFN-rich microenvironment of a T cell-inflamed tumor ultimately promotes mismatch between the neoepitope and T cell repertoires due to a shift from utilization of the constitutive proteasome to the immunoproteasome, thereby influencing tumor visibility (22). Furthermore, defects in β2-microglobulin expression can further impair antigen processing and display, affecting stable expression of MHC I molecules for their adequate surface expression and subsequent T cell recognition (23, 24). MHC class I haplotype loss or overall downregulation has been associated not only with altered tumor cell growth characteristics, but also facilitates evasion of immune surveillance (25). In melanoma, MHC class I internalization induced by BRAF V600E has also been described, suggesting another potential mechanism underlying the enhanced tumor visibility resulting from BRAF inhibitor therapy (9). Specific MHC class I loss has also been demonstrated in the evasion of T cell therapy for colorectal cancer (26). In addition, not all neoantigens bind MHC with high affinity, and the combinatorial match between neoantigen and MHC molecules expressed in the same cell determine how intrinsically immunogenic a neoantigen can be.

Studies of patient samples and *ex vivo* evidence strongly support the dominance of mutational neoantigens as targets for lymphocyte recognition of tumor, even in cancer types with lower overall mutational burden (10, 27–29). The importance of considering the available HLA sub-types and the T cell repertoire also present in the tumor, and their importance as major determinants of the tumor sub-clonal pruning that results from ongoing cycles of immune recognition, attack, and clearance, is now being appreciated (30). Computational methods exist to infer neoantigen expression and HLA binding characteristics from genomic and transcriptomic data (31, 32), and have been shown to act as a surrogate for treatment response in the context of checkpoint blockade immunotherapy in melanoma (10, 29). In fact, the sole presence of a more diverse array of HLA molecules (i.e., HLA heterozygosity), presumably linked to the ability to present a wider breadth of neoantigens, has recently been associated with increased survival in melanoma and lung cancer patients treated with immune checkpoint blockade (33). Knowledge of the mutational landscape of a tumor is thus of great importance to estimating the outcome of both targeted and immune therapies, however, measures of mutational and neoantigen burden alone do not predict immunotherapeutic outcome perfectly and results have been conflicting in separate cohorts (29, 30, 34). Similarly, though predictive approaches have been utilized to identify neoantigen candidates based on somatic mutations, these algorithms remain suboptimal, likely due to the numerous moderating factors described above (35). Accordingly, predictive approaches are now being paired with additional filters provided by proteasomal cleavage algorithms, as well as expression data to evaluate somatic mutations which are adequately expressed. A smaller number of neoantigen candidates can then be tested with autologous lymphocytes through molecular cloning of tandem minigenes comprising numerous expressed neoantigens (11).

## Tumor Immune “Susceptibility”

A tumor’s visibility to the immune system does not automatically imply its clearance, and numerous distinct factors can also influence its susceptibility to immune attack, which may be related to or completely independent of visibility.

In recent work, Chen and Mellman described the different immune infiltration profiles associated with response, which were classified as “inflamed,” “immune-excluded,” and “immune-desert” (36). Tumor immune susceptibility is inherently greater in patients of the “inflamed” type, where immune cells are present and capable of exerting their anti-tumor effects. Although immune visibility is critical to the establishment of an inflamed tumor microenvironment, the outcome of tumor inflammation can be influenced by a series of factors which build on a tumor’s visibility, such as chemokines, pro-inflammatory cytokines, and effector T cell density and function. Conversely, immunosuppressive cytokines and the presence of pro-tumor immune inhibitory cell types, such as tumor-associated (M2) macrophages, regulatory T cells (Treg), and myeloid-derived suppressor cells (MDSCs) can lead to development of an immune-desert tumor microenvironment, clearly detrimental to response.

Recently, an extensive genome-scale *in vitro* CRISPR/Cas9 screen revealed genes involved in antigen presentation and IFN-signaling to be most relevant to the ability of CD8 T cells to kill melanoma cells (37). IFN-γ signaling defects have been repeatedly implicated in cancer immunotherapy failure, including copy-number losses of IFN-γ pathway genes (principally *IFNGR1/2, IRF1*, and *JAK2*) in patients failing to respond to anti-CTLA-4 therapy (38). Loss-of-function mutations in *JAK1* and *JAK2* have also been described in the tumors of melanoma patients with either primary (39) or secondary (24) resistance to anti-PD-1 therapy. It must be noted that while tumoral inflammation appears a common if not necessary component of the anti-cancer immune response (regardless of therapeutic agent used), persistent activation of IFN-driven inflammatory signals adaptively leads to upregulation of inhibitory checkpoint molecules on lymphocytes and generation of an immunosuppressive microenvironment (40, 41). Thus, optimal immunotherapeutic outcomes may require more complex sequencing and/or intermittent dosing strategies than have yet been studied in patients.

In keeping with the concept of immune-inflamed and immune-excluded or immune-desert phenotypes described by Chen and Mellman, microenvironmental characteristics affecting lymphocyte entry and trafficking are critical to the efficacy of immunotherapy. Baseline lymphocytic infiltrate, particularly CD8 T cell density, is predictive of response to checkpoint inhibitor monotherapy (42), with early on treatment biopsies being more highly predictive of response than at baseline (43). Such “snapshots” of the immune infiltrate represent the combination over time of factors affecting T cell recruitment and T cell exclusion, such as a tumor cell-intrinsic activation of β-catenin (44). In fact, in work by Spranger and colleagues, it was demonstrated that the absence of tumor-derived β-catenin signaling allows production of CCL4, a chemokine which aids dendritic cell recruitment and thereby promotes T cell priming and anti-tumor responses (44). Furthermore, loss of expression of genes such as *PTEN* may influence the immune response through increased expression of immunosuppressive cytokines, such as VEGF and CCL2 (4). In fact, *PTEN* loss in melanoma patients was associated with progression on PD-1 blockade, possibly due to this mechanism, with CD8 T cell exclusion shown in regions of the tumor devoid of PTEN expression. Angiopoietic factors such as VEGF are frequently secreted by tumors and contribute to treatment failure (45). Pre-clinical models and translational studies of combined immune checkpoint blockade and anti-angiogenic agents suggest a potentially complex effect on tumor immunity, including beneficial effects on DC function and suppressive capacity of intra-tumoral MDSCs (46), enhanced anti-tumor humoral immunity (47), and increased lymphocyte trafficking and recruitment (47, 48).

Failure of spontaneous anti-tumor activity may largely be due to a dysfunctional “exhausted” T cell state associated with high expression of negative regulatory checkpoint molecules that are nonetheless amenable to treatment with modern checkpoint blockade immunotherapy (49). A more comprehensively inhibited T cell phenotype, typically with expression of numerous inhibitory checkpoint molecules including TIM-3, LAG-3, and others, may contribute to resistance to checkpoint inhibitor therapies in current clinical use (50). The presence of Treg as a key inhibitory factor on the anti-tumor response is, conversely, associated with poor response to checkpoint blockade.

## Complexities of Tumor Heterogeneity

That the majority of treatments for advanced cancers fail to produce curative outcomes is testament to the sheer diversity of cancer cell sub-populations present, limited in number only by the ability of our technologies to unravel their complexities at a molecular level. Heterogeneity of tumor cells, infiltrating immune cells, local vasculature, chemokine/cytokine gradients, and the underlying genetic basis for these variations are thus highly relevant to multiple aspects of immunotherapy efficacy. Tumoral heterogeneity influences both visibility and susceptibility of a tumor to immune attack, and has been described across cancer types (51–53).

Heterogeneity may arise from stochastic variation between cell sub-populations as cancer cells divide and accumulate mutations, or as a consequence of more plastic processes which shape cell state, gene expression, cellular function, and phenotype in response to prevailing selective processes over time or in different microenvironments (54–56). Heterogeneity may also arise as a direct consequence of sub-clonal immunoediting that occurs during both spontaneous and treatment-related anti-cancer immune responses, leading to non-uniform expression of target antigen (57) or essential antigen presentation machinery (25) across tumors.

The impact of tumor heterogeneity was recently highlighted in localized lung adenocarcinoma, demonstrating that a substantial proportion of tumor mutations are sub-clonal, i.e., restricted to regions of a tumor (58). This pattern extended to the neoantigens derived from these mutations, and patients with the highest proportion of sub-clonal neoantigens experienced shortened disease-free survival. Similar findings were seen when studying the T cell repertoire, where patients with the most heterogeneity in their T cell repertoire fared worst, highlighting the direct implications of genomic and immune heterogeneity on patient outcome (58).

Future treatment strategies will need to consider the effects of pre-existing tumoral heterogeneity as well as the adaptive treatment-induced changes that contribute to treatment failure. Furthermore, treatment strategies may also exert unique effects on tumor heterogeneity. In a recent melanoma study, prior therapy did not affect genomic inter-tumor heterogeneity whereas immune heterogeneity was more limited in patients previously treated with checkpoint blockade (52). As late stage patients become increasingly heavily pre-treated, the effects of these prior therapies on tumor heterogeneity will also need to be taken into consideration.

## Systemic and Environmental Influences on Response

**Figure 2:**
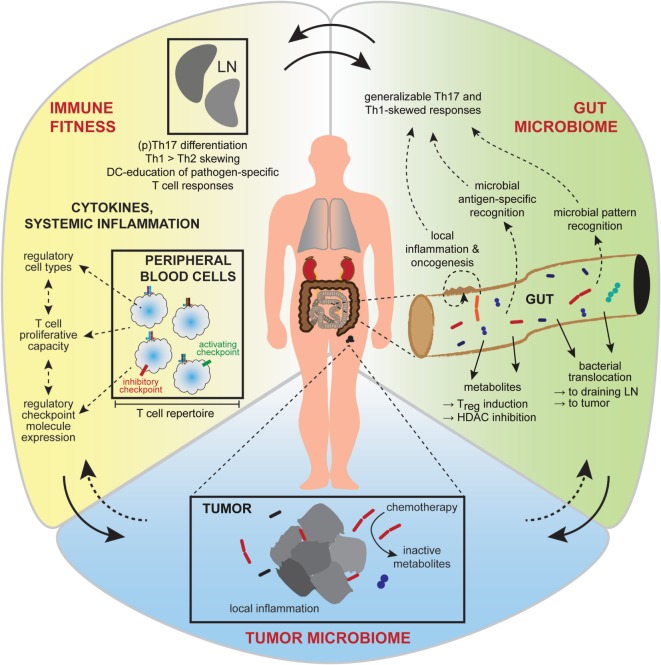
Interactions between the gut and intra-tumoral microbiota and the systemic immunity affect treatment outcome. Broader influences on tumor growth and responsiveness to immunotherapy are now realized, including contributions from the gut microbiome, the tumor microbiome, and systemic factors affecting general immune fitness. Interactions between these conceptual compartments are complex and incompletely understood. Gut microbiota (*green*) have diverse metabolic and antigen or pattern-molecule immunogenic effects on local gut inflammation, the effects of which contribute to local carcinogenesis or can become generalized to affect cancer growth and immunity at distant body sites. Favorable immune fitness (*yellow*), characterized by overall skewing toward cellular immune responses, a permissive cytokine milieu and activation-biased representation of anti-tumor, and regulatory immune cell types, is important for anti-tumor responses. The intra-tumoral microbiome (*blue*) is of emerging significance, having local inflammatory and metabolic effects that influence therapeutic sensitivity.

Although undeniably important, the metabolic, vascular, and immune dynamics active in the tumor microenvironment are only some of the contributing factors. It is now quite clear that overall host immunity as well as environmental influences (Figure 2) can shape therapeutic responses (59), and these factors will be discussed herein.

## Influence of Overall Immune Fitness

Effective anti-tumor immune responses require exposure of the tumor microenvironment to a wide network of innate and adaptive immune effector populations recruited from the systemic circulation. These cells must recognize and target tumor cells for elimination, based on the visibility factors described previously, in a critical process termed “immunosurveillance” (60). Three core phases of immunosurveillance have been described, spanning elimination of susceptible tumor cells through equilibrium (in which visibility-susceptibility mismatch or selective pruning of the most immune-susceptible tumor sub-clones leads to an anti-cancer stalemate), to escape (in which selection of low-visibility low-susceptibility tumor cells facilitates renewed tumor progression) (61). These dynamic phases occur spontaneously, but are undoubtedly influenced by exposure to immunotherapies, as shown in a parallel genomic and immune analysis of tumors from patients with advanced melanoma who received treatment with the anti-PD-1 agent nivolumab (30). In this study, clear patterns of mutational contraction and T cell clonal expansion occurred in what appeared to be a refocusing of the immune-cancer interaction and elimination of neoantigen-expressing sub-clones in patients who responded to therapy. While the specific cellular interactions that characterize each phase of immunosurveillance occur within the tumor microenvironment, the immune cells involved are sensitively dependent on adequate supply from the systemic compartment, and a prevailing immune phenotype conducive to anti-tumor activity (which may be therapeutically modifiable).

Recent work by Spitzer and colleagues provides key evidence supporting the direct relevance of systemic immune function to cancer immunotherapy (62). In this study, extensive high dimensional immune profiling using mass cytometry was performed in a MMTV-PyMT breast cancer mouse model to explore the dynamics of multiple immune cell populations in response to either effective, or ineffective, anti-cancer immunotherapy, in multiple body compartments. Many immune subsets were found to proliferate within the tumor microenvironment during the initiation of immune responses. Importantly, however, the proliferation of multiple immune cell populations, including B cells, NK cells, dendritic cells, and effector/memory T cells, during active tumor rejection was primarily sustained in secondary lymphoid organs and not the intra-tumoral compartment, indicating the significance of the systemic compartment to maintenance of effective anti-cancer immune responses (62).

Myeloid-derived suppressor cells have garnered much interest in recent years for their immune-suppressive capabilities across various cancer types. MDSCs are a phenotypically heterogeneous group of cells comprised of immature myeloid cells, and broadly divided into monocytic and granulocytic sub-types (63). MDSCs have a potent ability to suppress T cell responses through numerous specific mechanisms in lymphoid organs, such as production of indoleamine 2,3 dioxygenase or arginase-1, which locally deplete crucial amino acids such as tryptophan and arginine, thereby rendering T cells functionally anergic. MDSCs may also inhibit T cell responses through production of immunosuppressive cytokines including TGF-β and IL-10, or generation of reactive oxygen species (ROS) (64). Because of this ability to inhibit T cell responses and promote tumor development, MDSCs have been suggested to be a key therapeutic target in cancer. Though MDSCs are present in the tumor microenvironment and tend to increase with cancer development, their characterization in the periphery has become an area of intense investigation, including studies of their relationship to clinicopathologic attributes and patient outcome. Overall, MDSCs are generally more abundant in the peripheral blood of cancer patients compared with healthy subjects. However, their frequencies also increase from early stage to late stage disease and with higher histological grade. These trends have been observed in numerous histologies such as renal cell cancer (65), colorectal carcinoma (66), melanoma (67), as well as gastric cancer (68). Interestingly, higher frequencies of MDSCs in the periphery have also been predictive of patient relapse in breast cancer (69), melanoma (67, 70), differentiated thyroid cancer (71), glioblastoma (72), head and neck squamous cell carcinoma (73), pancreatic cancer (74), prostate cancer (75), and renal cell carcinoma (76). Their increased frequencies in the circulation have also been tied to the development of metastases in melanoma and colorectal cancer (66). Together, the somewhat graded relationship between MDSC abundance and cancer progression, including metastasis, suggests that circulating MDSCs at least reflect the immunosuppressive status of the tumor microenvironment, and likely fulfill a more direct role in the development of a systemically suppressed immune response. Unfortunately, inconsistency in the classification and functional characterization of MDSCs has limited our ability to accurately enumerate and isolate them, highlighting some of the challenges in translating the therapeutic targeting of MDSCs to the clinic.

Despite the importance of immune fitness in therapeutic response, our ability to assess anti-tumor immune responses in the peripheral circulation remains somewhat limited to date and uncertainties remain regarding the contributions made by immune populations at different times, and at different sites. Insofar as upregulation of inhibitory checkpoint molecules is associated with prior antigen exposure and activation, PD-1 expression on circulating T cells was found to enrich for neoantigen-specific T cells in the peripheral blood of melanoma patients (77). However, data showing that higher clonality of the T cell repertoire resident within tumors before therapy was associated with better outcome to PD-1 blockade (34, 42) suggests that much of the relevant effector immune population is already intra-tumoral even before therapy. Thus, the proportion of tumor-specific T cells present in the general circulation at any instant may be relatively low, limiting what can be concluded about therapeutic response from examination of the periphery in isolation, with currently available tools. Looking even more generally, the broad functional status of the adaptive immune system, defined as Th1-, Th17-, or Th2-skewing of circulating immune cells, may be more reliably associated with the prevalent immunophenotype of tumor-infiltrating lymphocytes (TIL), reflecting the likelihood of effective cytotoxic rather than tolerogenic cellular immune outcomes.

## The Gut Microbiome Modulates Cancer Development, Immunity and Response

Systemic immunity is shaped by interactions with our environment, and there is now clear evidence that the gut microbiome contributes to the establishment and maintenance of systemic immune tone (78, 79). As the largest commensal microbial community (80, 81), the gut microflora has been extensively studied as a trigger for local inflammation in non-malignant conditions such as inflammatory bowel disease (82, 83). Overall, these microbes present a significant challenge to the host’s immune defenses, which must regulate tolerance to beneficial microbes while guarding against harmful pathogens. Moreover, recent evidence suggests that the gut microbiome plays a significant role in cancer development and response to cancer therapy as discussed in the following sections.

Gut microbiota represent a double-edged sword that can promote or inhibit cancer development, with both individual bacterial taxa and overall bacterial dysbiosis implicated in oncogenic initiation and progression. *Helicobacter pylori*–particularly those containing the virulence factor cagA–have been extensively characterized as an oncogenic initiator, particularly in gastric adenocarcinoma (84). In a murine model, a potentially pathogenic enterotoxigenic *Bacteroides fragilis*-induced a STAT3-dependent, Th17-mediated colitis associated with colonic tumor formation in pre-disposed mice; colitis and tumor formation were prevented by administration of blocking antibodies to IL-17 and IL-23 (85). *Escherichia coli* strains may harbor the polyketide synthase genomic island (pks), which encodes a genotoxin called colibactin that induces DNA damage in murine colonocytes. Furthermore, *E. coli* that are pks+ are more frequently identified in colon cancer patients compared with healthy controls (86, 87). On the other hand, bacteria can also be tumor suppressive as demonstrated by *Butyrivibrio fibrisolvens*, which resulted in colorectal tumor attenuation by producing copious amounts of butyrate in the presence of a high-fiber diet in a rat-azoxymethane model. Importantly, population-based metagenomic analyses in colon cancer patients have also revealed differential enrichment of bacterial taxa (especially *Fusobacterium)* in colorectal tumors when compared with controls (88–91).

As opposed to microbial taxa that drive oncogenesis in a highly penetrant manner, the relationship between dysbiosis and cancer is more complex and multifactorial. Dysbiosis is influenced by several extraneous factors including diet, antibiotic use, and smoking (92). In pre-clinical models of colon carcinoma, both germ-free status and antibiotic treatment have been found to be associated with reduced incidence of tumors (93–96). A dysbiotic microbiota can also influence cancer development at distant sites such as the liver (97) and pancreas through pro-inflammatory microbe-associated molecular patterns and metabolites released into the systemic circulation (98).

Several murine studies have established a clear requirement for a diverse and intact intestinal microbiota to achieve optimal response to distinct cancer treatment modalities through effects on both the innate and adaptive arms of the immune system. The gut microbiome is now implicated in modulating responses across a wide range of cancer therapies, including intra-tumoral therapy (99), chemotherapy (99, 100), and immune checkpoint blockade (101–103). Attempts to define the mechanisms underlying microbial associations with the efficacy of cancer therapies have revealed both microbe-specific and microbe-agnostic influences (Figure 2). For instance, administration of LPS alone, in the presence of its cognate pattern recognition receptor, largely restored the efficacy of intralesional immunotherapy administered to either germ-free or antibiotic-ablated mice implanted with melanoma or colon cancer (99). In these models, systemic microbial priming of myeloid lineage cells appeared essential to their subsequent intra-tumoral accumulation and production of pro-inflammatory and chemotactic cytokines or ROS, without which an effective secondary T cell infiltrate could not be recruited. Interestingly, responses to chemotherapy may be facilitated by disruption of the integrity of the gut epithelial barrier with subsequent bacterial translocation of *Enterococcus hirae* and *Lactobacillus johnsonii* and priming of immune responses demonstrated in a murine model (100). Bacteria such as *E. hirae* and *Barnesiella intestinihominis* can also affect immune responses directly at the tumor site, with depletion of intra-tumoral Treg and accumulation of γδT cells, respectively (104).

The initial demonstration that several bacterial taxa were associated with response to immune checkpoint blockade came from murine studies, which implicated *Bacteroides thetaiotaomicron* and *B. fragilis* in the case of CTLA-4 blockade (101) and *Bifidobacterium*, in the case of PD-L1 blockade (103). These studies were followed by several analyses of patient cohorts that confirm a clear role for the gut microbiome in modulating responses to immune checkpoint blockade across cancer types (102, 105). A reciprocal relationship between the mid-level taxa Clostridiales (favorable) and Bacteroidales (unfavorable) and likelihood of response were recently reported in a large cohort of melanoma patients (102). Importantly, fecal microbial transplantation into germ-free mice using stool from responding patients resulted in enhanced tumor control when compared with donor stool from non-responding patients. Other population-level studies have also reported similar findings with regards to over-representation of the *Faecalibacterium* genus of the Clostridiales order in pre-treatment samples of responders to anti-CTLA-4 and combination anti-CTLA-4 plus anti-PD-1 immunotherapies (106, 107). In another study, *Bifidobacterium* was also found to be enriched in melanoma patients who were responding positively to anti-PD-1 therapy, analogous to earlier results implicating this taxon in a murine model of PD-L1 (105).

Interestingly, the bacterial taxa identified in these human studies differ somewhat from those identified in murine experiments and even across the patient cohorts, suggesting the need for additional studies to address the significance of geographical and other variables influencing microbiome composition and response, and to confirm unifying taxa or functionalities to take forward to clinical development. These studies also imply that administration of single bacterial species may not be reliably effective in modulating responses to immunotherapy.

Our understanding of the influence of the gut microbiome on immunity and therapeutic responses is evolving, and it is evident that micro-organisms may share functionalities that convey immunomodulatory properties that are not immediately evident from taxonomic discovery. Indeed, several investigators have demonstrated a key integrative role of microbial metabolites such as short chain fatty acids (SCFAs) produced by microbial fermentation of undigested complex carbohydrates in mediating the effect of commensal bacteria on immune tone (108, 109). In fact, a significant proportion of metabolites found in human plasma are microbiome-derived and can affect immune cells by influencing their metabolic circuits or engaging with metabolite-specific receptors. SCFAs act as signaling molecules, by inhibiting histone deacetylases (HDAC). Their action on lymphocytes and neutrophils is mediated *via* the blockade of NF-κB and the subsequent downstream production of pro-inflammatory TNF (110). Importantly, SCFAs promote homeostasis by regulating the size and function of the colonic FoxP3+ Treg pool in an HDAC-dependent manner (108, 109). SCFAs can also exert a regulatory effect by signaling through G protein-coupled receptors, resulting in the limitation of neutrophil chemotaxis (111) and expansion of Treg function (112). Therefore, it is also not surprising that these metabolites can directly affect cancer cells by impacting immunosurveillance (113).

At the population-level, numerous studies have identified high diversity of the gut microbiota as being associated with improved survival outcomes in allogeneic hematopoietic stem cell transplantation patients, together with relatively lower rates of graft-vs-host disease (114, 115). Consistent with these findings, a beneficial effect was also reported in the context of anti-PD-1 therapy in melanoma patients, wherein high diversity of the gut microbiome at baseline was found to be associated with significantly improved progression-free survival rates (102). Importantly, a similar provocative observation was also made in the context of lung, renal, and bladder cancer patients where disruption of microbial diversity of the gut was found to have a detrimental effect on the efficacy of immune checkpoint blockade (116). In these studies, the authors demonstrated that antibiotic usage shortly before, during or shortly after the initiation of treatment with immune checkpoint blockade was associated with significantly reduced progression-free and overall survival. In addition, metagenomic sequencing implicated the species *Akkermansia muciniphila* to be abundant in responders to anti-PD-1, and capable of restoring its efficacy in germ-free mice transplanted with non-responder patient stool (116).

## The Tumor Microbiome and Response

In addition to the gut microbiome, bacteria within tumors themselves may influence cancer development as well as therapeutic responses. This has been studied most extensively in colorectal cancer, where certain bacterial taxa such as *Fusobacterium nucleatum* and *Streptococcus bovis* have been associated with primary tumors (88, 117, 118) as well as in metastatic sites (119). *Fusobacterium*, specifically *F. nucleatum*, is enriched in colorectal carcinomas relative to normal colonic tissues (88, 89). The abundance of *F. nucleatum* within colon cancer tissues inversely correlates with recurrence-free survival, and appears adversely prognostic, comparable to increasing AJCC stage (120). Profiling the fecal microbiota or screening for known microbial markers associated with colon carcinogenesis such as the genotoxin colibactin and its encoding genotoxin cluster pks found in oncogenic *E. coli*, may provide a novel strategy for population screening, and may additionally provide clues as to the underlying mechanisms driving such microbial associations with the accumulated genetic damage that characterizes malignancy (121, 122). In this regard, detailed study of the molecular products of the pks island confirm that colibactin directly damages DNA (122) and mediates pks+ *E. coli* promotion of tumor formation in a murine *Apc^Min/+^*; *Il10^−/−^* model (123). While chronic DNA damage is undesirable, it is clear that a high mutational burden may in fact be beneficial in patients receiving checkpoint blockade immunotherapy, thus the relevance of such microbial genotoxicity on contemporary treatment outcomes warrants particular study. Furthermore, certain pathogenic taxa are linked to enrichment of inflammatory and DNA damage-response pathways in tumor transcriptomes, together with a distinct methylation and microsatellite instability profile (124). In light of these data, it is probable that the genomic and immune characteristics of intestinal tumors may be sensitively linked to the geographic microbial niches in which they arise.

Importantly, therefore, bacterial-associated molecular alterations in tumors span genomic, epigenetic and immune domains with the immunomodulatory effects of tumor-associated microbes appearing equally as diverse as the observed genomic and biochemical effects. In the context of *Fusobacterium*, direct molecular interaction between the bacterial Fap2 protein and TIGIT present on human NK cells contributes to tumor immune evasion; this interaction was shown to inhibit TIGIT-mediated activation of NK cell killing of colon adenocarcinoma cells, and was more generally suppressive of TIL (NK and T cells) killing using patient-derived matched TIL and tumor cells from melanoma patients (125). Furthermore, *F. nucleatum* appears to promote colorectal cancer cell chemoresistance to select agents in a complex multi-step sequence of molecular changes involving TLR4 and MYD88 activation and culminating in activation of autophagy (120). Colonic Th17-responses represent a common and partially unifying feature of many microbiota-associated local and systemic inflammatory states and have been associated with poor-responses to anti-cancer therapies (100). As previously noted, colon cancer formation associated with enterotoxigenic *B. fragilis* has been shown to involve Stat3-driven colitis and induction of a Th17 response that was prevented by IL-17 and IL-23 blockade in mouse models (85). *B. fragilis*-induced Th17-driven tumorigenesis involves the promotion of a suppressive myeloid environment characterized by monocytic MDSCs and consequently suppressed T cell proliferation (126). While Th17 skewing may thus form a major contribution to carcinogenesis and influence systemic chemotherapeutic responses in non-intestinal tumor models, these same mechanisms, and the specific effect of *B. fragilis*, were required for the efficacy of anti-CTLA-4 checkpoint blockade immunotherapy (101). This emphasizes the new complexity that has arisen with the advent of checkpoint molecule-targeted immunotherapies, for which the distinction between “favorable” and “unfavorable” microbiota is potentially reversed depending on whether the context at hand is one of cancer *development*, or immunotherapy-based *treatment* of an already-established cancer.

In addition to their roles in carcinogenesis and immunomodulation, intra-tumoral bacteria may also modulate responses to cytotoxic cancer therapy. *Mycoplasma hyorhinis*, which was associated with fibroblasts in the tumor microenvironment in pancreatic ductal adenocarcinoma tumors, was found to be able to direct drug metabolism and diminish the efficacy of gemcitabine. Further analyses of bacterial genes implicated the enzyme cytidine deaminase contained in the Gammaproteobacteria class to be necessary and sufficient to mediate conversion of gemcitabine to its inactive form, by expression of the long isoform of the enzyme cytidine deaminase, in a colon cancer murine model (127). Depletion of bacteria within the tumor and a robust anti-cancer response to gemcitabine were noted in tumor-bearing mice treated with the combination of gemcitabine and ciprofloxacin delivered directly to the tumor site. A high prevalence of Gammaproteobacteria was subsequently identified in pancreatic adenocarcinoma samples from patients, and retained the ability to confer gemcitabine resistance after *ex vivo* co-culture with colon cancer cell lines (127). How these intra-tumoral bacteria interact with infiltrating immune cells has not been completely elucidated.

Direct spatial microbe-tumor interactions are not only relevant to gastrointestinal cancers. Recent analyses of the microbiota present in breast cancer tissue compared with normal breast tissue revealed distinct microbial communities, driven by a lower abundance of *Methylobacterium* in cancerous tissues (128). The authors performed a parallel comparison of microbiota present at distant sites, with the provocative finding that urinary microbiota were also distinct between cancer patients and controls, even after correction for menopausal status. The mechanisms through which bacteria induce carcinogenesis may include induction of inflammation (85), altered cell signaling (129) and inhibition of T cell and natural killer cell responses (125), however the precise role of intra-tumoral bacteria in carcinogenesis across tumor types remains incompletely elucidated.

## Conclusion

Our future conceptualization of what matters for good outcomes to cancer immunotherapy requires a thoroughly integrated understanding of what contributes to cancer formation and immune evasion in the first place. Tumor mutational load provides an instructive example; while a high tumor mutational load is clearly important for response to checkpoint blockade immunotherapy, it still lacks adequate negative predictive value to be trusted in the clinic, and performs poorly for combination checkpoint blockade. If highly mutated tumors were truly (simplistically) vulnerable to immune clearance, it should be considered remarkable that they are observed at all. More likely, the snapshot measurement of mutational load is limited by numerous other factors, such as the neoantigen characteristics of the available pool of mutations, genomic methylation and transcriptome patterns, the intrinsic immunomodulatory effects of the tumor over time, and the extrinsic immunomodulatory effects of the patient’s environment and microbiota. Recent research highlights the critical need to model these interactions systematically and dynamically, taking adaptive evolution into consideration rather than relying on static measurements at single moments in time.

The importance of integrated biomarker models in cancer is highlighted well by the rapidly expanding interest in the study of the commensal microbiota in the context of cancer development and progression. Microbes influence the response to traditional cytotoxic agents through a diverse combination of effects on cellular metabolism, local pharmacokinetics, and could plausibly affect bioavailability of orally administered agents which are increasingly common in the era of targeted therapy, although this remains to be studied. Perhaps of greater significance, local interactions between specific bacteria and host tissues contribute to locoregional inflammation and carcinogenesis. Molecular interactions with pattern receptors (e.g., TLR4 and downstream MYD88), and immunosuppressive signals mediated by engagement with cell surface inhibitory molecules (e.g., TIGIT) or elaboration of suppressive cytokines (e.g., VEGF and CCL2) result in immune evasion that likely contributes to ineffective immunosurveillance of nascent, developing and established neoplasia.

It is not yet fully know to what extent the immunomodulatory effects of cancer-associated microbes may influence cancer immunotherapies, however, it is highly likely that these effects will not be consistent. For instance, immunosuppressive mechanisms such as MDSC-induction by *B. fragilis* suggest a negative impact on anti-cancer immunotherapy treatment response, however, the often simultaneous induction of Th17- and Th1-biased systemic immunity by the same organism(s) appears beneficial to checkpoint blockade immunotherapy response. This apparent contradiction will likely prove to be a complex and difficult issue to resolve as the field progresses, particularly as it relates to how best–or how safely–to manipulate the gut microbial composition to optimize treatment outcomes, toxicity, and long-term general health.

As more mechanisms are identified by which microbes directly influence tumor genomics, it will be important to evaluate whether the commonly observed EMT-like processes that accompany tumor progression involve a feed-forward loop precipitated by tumor-induced dysbiosis and subsequent microbe-directed epigenetic reprogramming of tumor cells. Another important issue that urgently requires attention is the relative significance of *individual* microbial taxa as opposed to unifying functional or metabolic characteristics of *multiple* taxa, in cancer development and treatment response. Metabolomic and whole genome shotgun sequencing studies are underway to address this, and will be highly relevant to the identification of the most readily targeted predisposing, permissive and perpetuating factors in cancer microbiology; it may be that critical intermediary metabolites or activated metabolic pathways prove to be most amenable to therapeutic modification. Further study is also required to integrate the significance of the microbiota with what is already known about the influence of lifestyle factors on cancer outcomes. Consideration should be made to the impact of duration, mode and type of microbiota “exposure” when integrating the microbiome into the new cancer-immunity model. As we continue to develop a deeper understanding of the myriad factors impacting cancer immunotherapy response, we will undoubtedly develop and refine new therapeutic strategies for maximal patient benefit.

## Author Contributions

All authors conceived, wrote, reviewed, and approved the final manuscript.

## Conflict of Interest Statement

MA and AR report no relevant conflict of interest or financial disclosures. VG and JW are inventors on a US patent application (PCT/US17/53717) submitted by The University of Texas MD Anderson Cancer Center that covers methods to enhance checkpoint blockade therapy by the microbiome. JW has honoraria from speakers’ bureau of Dava Oncology, Bristol-Myers Squibb, and Illumina, and is an advisory board member for GlaxoSmithKline, Novartis, and Roche/Genentech.

## References

[1] Curtin JA Busam K Pinkel D Bastian BC. Somatic activation of KIT in distinct subtypes of melanoma. J Clin Oncol (2006) 24(26):4340–6. 10.1200/JCO.2006.06.2984 16908931

[2] Forbes SA Bhamra G Bamford S Dawson E Kok C Clements J The catalogue of somatic mutations in cancer (COSMIC). Curr Protoc Hum Genet (2008) Chapter 10:Unit 10 1. 10.1002/0471142905.hg1011s57 18428421 PMC2705836

[3] Frederick DT Piris A Cogdill AP Cooper ZA Lezcano C Ferrone CR BRAF inhibition is associated with enhanced melanoma antigen expression and a more favorable tumor microenvironment in patients with metastatic melanoma. Clin Cancer Res (2013) 19(5):1225–31. 10.1158/1078-0432.CCR-12-1630 23307859 PMC3752683

[4] Peng W Chen JQ Liu C Malu S Creasy C Tetzlaff MT Loss of PTEN promotes resistance to T cell-mediated immunotherapy. Cancer Discov (2016) 6(2):202–16. 10.1158/2159-8290.CD-15-0283 26645196 PMC4744499

[5] Kono M Dunn IS Durda PJ Butera D Rose LB Haggerty TJ Role of the mitogen-activated protein kinase signaling pathway in the regulation of human melanocytic antigen expression. Mol Cancer Res (2006) 4(10):779–92. 10.1158/1541-7786.MCR-06-0077 17050671

[6] Boni A Cogdill AP Dang P Udayakumar D Njauw CN Sloss CM Selective BRAFV600E inhibition enhances T-cell recognition of melanoma without affecting lymphocyte function. Cancer Res (2010) 70(13):5213–9. 10.1158/0008-5472.CAN-10-0118 20551059

[7] Wilmott JS Long GV Howle JR Haydu LE Sharma RN Thompson JF Selective BRAF inhibitors induce marked T-cell infiltration into human metastatic melanoma. Clin Cancer Res (2012) 18(5):1386–94. 10.1158/1078-0432.CCR-11-2479 22156613

[8] Donia M Fagone P Nicoletti F Andersen RS Hogdall E Straten PT BRAF inhibition improves tumor recognition by the immune system: potential implications for combinatorial therapies against melanoma involving adoptive T-cell transfer. Oncoimmunology (2012) 1(9):1476–83. 10.4161/onci.21940 23264894 PMC3525603

[9] Bradley SD Chen Z Melendez B Talukder A Khalili JS Rodriguez-Cruz T BRAFV600E Co-opts a conserved MHC class I internalization pathway to diminish antigen presentation and CD8+ T-cell recognition of melanoma. Cancer Immunol Res (2015) 3(6):602–9. 10.1158/2326-6066.CIR-15-0030 25795007 PMC4457616

[10] Snyder A Makarov V Merghoub T Yuan J Zaretsky JM Desrichard A Genetic basis for clinical response to CTLA-4 blockade in melanoma. N Engl J Med (2014) 371(23):2189–99. 10.1056/NEJMoa1406498 25409260 PMC4315319

[11] Tran E Turcotte S Gros A Robbins PF Lu YC Dudley ME Cancer immunotherapy based on mutation-specific CD4+ T cells in a patient with epithelial cancer. Science (2014) 344(6184):641–5. 10.1126/science.1251102 24812403 PMC6686185

[12] Woods K Pasam A Jayachandran A Andrews MC Cebon J. Effects of epithelial to mesenchymal transition on T cell targeting of melanoma cells. Front Oncol (2014) 4:367. 10.3389/fonc.2014.00367 25566505 PMC4269118

[13] Konieczkowski DJ Johannessen CM Abudayyeh O Kim JW Cooper ZA Piris A A melanoma cell state distinction influences sensitivity to MAPK pathway inhibitors. Cancer Discov (2014) 4(7):816–27. 10.1158/2159-8290.CD-13-0424 24771846 PMC4154497

[14] Alexandrov LB Nik-Zainal S Wedge DC Aparicio SA Behjati S Biankin AV Signatures of mutational processes in human cancer. Nature (2013) 500(7463):415–21. 10.1038/nature12477 23945592 PMC3776390

[15] Lawrence MS Stojanov P Polak P Kryukov GV Cibulskis K Sivachenko A Mutational heterogeneity in cancer and the search for new cancer-associated genes. Nature (2013) 499(7457):214–8. 10.1038/nature12213 23770567 PMC3919509

[16] Goodman AM Kato S Bazhenova L Patel SP Frampton GM Miller V Tumor mutational burden as an independent predictor of response to immunotherapy in diverse cancers. Mol Cancer Ther (2017) 16(11):2598–608. 10.1158/1535-7163.MCT-17-0386 28835386 PMC5670009

[17] Rizvi NA Hellmann MD Snyder A Kvistborg P Makarov V Havel JJ Cancer immunology. Mutational landscape determines sensitivity to PD-1 blockade in non-small cell lung cancer. Science (2015) 348(6230):124–8. 10.1126/science.aaa1348 25765070 PMC4993154

[18] Le DT Uram JN Wang H Bartlett BR Kemberling H Eyring AD PD-1 blockade in tumors with mismatch-repair deficiency. N Engl J Med (2015) 372(26):2509–20. 10.1056/NEJMoa1500596 26028255 PMC4481136

[19] Johnson DB Frampton GM Rioth MJ Yusko E Xu Y Guo X Targeted next generation sequencing identifies markers of response to PD-1 blockade. Cancer Immunol Res (2016) 4(11):959–67. 10.1158/2326-6066.CIR-16-0143 27671167 PMC5134329

[20] Rizvi H Sanchez-Vega F La K Chatila W Jonsson P Halpenny D Molecular determinants of response to anti-programmed cell death (PD)-1 and anti-programmed death-ligand 1 (PD-L1) blockade in patients with non-small-cell lung cancer profiled with targeted next-generation sequencing. J Clin Oncol (2018) 36(7):633–41. 10.1200/JCO.2017.75.3384 29337640 PMC6075848

[21] Gandara DR Kowanetz M Mok TSK Rittmeyer A Fehrenbacher L Fabrizio D editors. Abstract 1295O: blood-based biomarkers for cancer immunotherapy: tumor mutational burden in blood (bTMB) is associated with improved atezolizumab (atezo) efficacy in 2L+ NSCLC (POPLAR and OAK). ESMO congress 2017. Ann Oncol (2017) 28(suppl 5):460 10.1093/annonc/mdx380

[22] Woods K Knights AJ Anaka M Schittenhelm RB Purcell AW Behren A Mismatch in epitope specificities between IFNgamma inflamed and uninflamed conditions leads to escape from T lymphocyte killing in melanoma. J Immunother Cancer (2016) 4:10 10.1186/s40425-016-0111-7 26885372 PMC4754849

[23] D’Urso CM Wang ZG Cao Y Tatake R Zeff RA Ferrone S. Lack of HLA class I antigen expression by cultured melanoma cells FO-1 due to a defect in B2m gene expression. J Clin Invest (1991) 87(1):284–92. 10.1172/JCI114984 1898655 PMC295046

[24] Zaretsky JM Garcia-Diaz A Shin DS Escuin-Ordinas H Hugo W Hu-Lieskovan S Mutations associated with acquired resistance to PD-1 blockade in melanoma. N Engl J Med (2016) 375(9):819–29. 10.1056/NEJMoa1604958 27433843 PMC5007206

[25] Garrido C Paco L Romero I Berruguilla E Stefansky J Collado A MHC class I molecules act as tumor suppressor genes regulating the cell cycle gene expression, invasion and intrinsic tumorigenicity of melanoma cells. Carcinogenesis (2012) 33(3):687–93. 10.1093/carcin/bgr318 22219178

[26] Tran E Robbins PF Lu YC Prickett TD Gartner JJ Jia L T-cell transfer therapy targeting mutant KRAS in cancer. N Engl J Med (2016) 375(23):2255–62. 10.1056/NEJMoa1609279 27959684 PMC5178827

[27] Lennerz V Fatho M Gentilini C Frye RA Lifke A Ferel D The response of autologous T cells to a human melanoma is dominated by mutated neoantigens. Proc Natl Acad Sci U S A (2005) 102(44):16013–8. 10.1073/pnas.0500090102 16247014 PMC1266037

[28] Tran E Ahmadzadeh M Lu YC Gros A Turcotte S Robbins PF Immunogenicity of somatic mutations in human gastrointestinal cancers. Science (2015) 350(6266):1387–90. 10.1126/science.aad1253 26516200 PMC7445892

[29] Van Allen EM Miao D Schilling B Shukla SA Blank C Zimmer L Genomic correlates of response to CTLA-4 blockade in metastatic melanoma. Science (2015) 350(6257):207–11. 10.1126/science.aad0095 26359337 PMC5054517

[30] Riaz N Havel JJ Makarov V Desrichard A Urba WJ Sims JS Tumor and microenvironment evolution during immunotherapy with nivolumab. Cell (2017) 171(4):934–49 e15. 10.1016/j.cell.2017.09.028 29033130 PMC5685550

[31] Liu C Yang X Duffy B Mohanakumar T Mitra RD Zody MC ATHLATES: accurate typing of human leukocyte antigen through exome sequencing. Nucleic Acids Res (2013) 41(14):e142. 10.1093/nar/gkt481 23748956 PMC3737559

[32] Hoof I Peters B Sidney J Pedersen LE Sette A Lund O NetMHCpan, a method for MHC class I binding prediction beyond humans. Immunogenetics (2009) 61(1):1–13. 10.1007/s00251-008-0341-z 19002680 PMC3319061

[33] Chowell D Morris LGT Grigg CM Weber JK Samstein RM Makarov V Patient HLA class I genotype influences cancer response to checkpoint blockade immunotherapy. Science (2018) 359(6375):582–. 10.1126/science.aao4572 29217585 PMC6057471

[34] Roh W Chen PL Reuben A Spencer CN Prieto PA Miller JP Integrated molecular analysis of tumor biopsies on sequential CTLA-4 and PD-1 blockade reveals markers of response and resistance. Sci Transl Med (2017) 9(379):eaah3560 10.1126/scitranslmed.aah3560 28251903 PMC5819607

[35] Nielsen M Lundegaard C Blicher T Lamberth K Harndahl M Justesen S NetMHCpan, a method for quantitative predictions of peptide binding to any HLA-A and -B locus protein of known sequence. PLoS One (2007) 2(8):e796. 10.1371/journal.pone.0000796 17726526 PMC1949492

[36] Chen DS Mellman I. Elements of cancer immunity and the cancer-immune set point. Nature (2017) 541(7637):321–30. 10.1038/nature21349 28102259

[37] Patel SJ Sanjana NE Kishton RJ Eidizadeh A Vodnala SK Cam M Identification of essential genes for cancer immunotherapy. Nature (2017) 548(7669):537–42. 10.1038/nature23477 28783722 PMC5870757

[38] Gao J Shi LZ Zhao H Chen J Xiong L He Q Loss of IFN-gamma pathway genes in tumor cells as a mechanism of resistance to anti-CTLA-4 therapy. Cell (2016) 167(2):397–404 e9. 10.1016/j.cell.2016.08.069 27667683 PMC5088716

[39] Shin DS Zaretsky JM Escuin-Ordinas H Garcia-Diaz A Hu-Lieskovan S Kalbasi A Primary resistance to PD-1 blockade mediated by JAK1/2 mutations. Cancer Discov (2017) 7(2):188–201. 10.1158/2159-8290.CD-16-1223 27903500 PMC5296316

[40] Benci JL Xu B Qiu Y Wu TJ Dada H Twyman-Saint Victor C Tumor interferon signaling regulates a multigenic resistance program to immune checkpoint blockade. Cell (2016) 167(6):1540–54.e12. 10.1016/j.cell.2016.11.022 27912061 PMC5385895

[41] Sharma P Hu-Lieskovan S Wargo JA Ribas A. Primary, adaptive, and acquired resistance to cancer immunotherapy. Cell (2017) 168(4):707–23. 10.1016/j.cell.2017.01.017 28187290 PMC5391692

[42] Tumeh PC Harview CL Yearley JH Shintaku IP Taylor EJ Robert L PD-1 blockade induces responses by inhibiting adaptive immune resistance. Nature (2014) 515(7528):568–71. 10.1038/nature13954 25428505 PMC4246418

[43] Chen PL Roh W Reuben A Cooper ZA Spencer CN Prieto PA Analysis of immune signatures in longitudinal tumor samples yields insight into biomarkers of response and mechanisms of resistance to immune checkpoint blockade. Cancer Discov (2016) 6(8):827–37. 10.1158/2159-8290.CD-15-1545 27301722 PMC5082984

[44] Spranger S Bao R Gajewski TF Melanoma-intrinsic beta-catenin signalling prevents anti-tumour immunity. Nature (2015) 523(7559):231–5. 10.1038/nature14404 25970248

[45] Johnson BF Clay TM Hobeika AC Lyerly HK Morse MA. Vascular endothelial growth factor and immunosuppression in cancer: current knowledge and potential for new therapy. Expert Opin Biol Ther (2007) 7(4):449–60. 10.1517/14712598.7.4.449 17373897

[46] Du Four S Maenhout SK Niclou SP Thielemans K Neyns B Aerts JL. Combined VEGFR and CTLA-4 blockade increases the antigen-presenting function of intratumoral DCs and reduces the suppressive capacity of intratumoral MDSCs. Am J Cancer Res (2016) 6(11):2514–31. 27904768 PMC5126270

[47] Wu X Giobbie-Hurder A Liao X Lawrence D McDermott D Zhou J VEGF neutralization plus CTLA-4 blockade alters soluble and cellular factors associated with enhancing lymphocyte infiltration and humoral recognition in melanoma. Cancer Immunol Res (2016) 4(10):858–68. 10.1158/2326-6066.CIR-16-0084 27549123 PMC5050160

[48] Shrimali RK Yu Z Theoret MR Chinnasamy D Restifo NP Rosenberg SA. Antiangiogenic agents can increase lymphocyte infiltration into tumor and enhance the effectiveness of adoptive immunotherapy of cancer. Cancer Res (2010) 70(15):6171–80. 10.1158/0008-5472.CAN-10-0153 20631075 PMC2912959

[49] Daud AI Loo K Pauli ML Sanchez-Rodriguez R Sandoval PM Taravati K Tumor immune profiling predicts response to anti-PD-1 therapy in human melanoma. J Clin Invest (2016) 126(9):3447–52. 10.1172/JCI87324 27525433 PMC5004965

[50] Koyama S Akbay EA Li YY Herter-Sprie GS Buczkowski KA Richards WG Adaptive resistance to therapeutic PD-1 blockade is associated with upregulation of alternative immune checkpoints. Nat Commun (2016) 7:10501. 10.1038/ncomms10501 26883990 PMC4757784

[51] Albino AP Lloyd KO Houghton AN Oettgen HF Old LJ. Heterogeneity in surface antigen and glycoprotein expression of cell lines derived from different melanoma metastases of the same patient. Implications for the study of tumor antigens. J Exp Med (1981) 154(6):1764–78. 10.1084/jem.154.6.1764 6976407 PMC2186539

[52] Reuben A Spencer CN Prieto PA Gopalakrishnan V Reddy SM Miller JP Genomic and immune heterogeneity are associated with differential responses to therapy in melanoma. NPJ Genom Med (2017) 2:10. 10.1038/s41525-017-0013-8 28819565 PMC5557036

[53] Morris LG Riaz N Desrichard A Senbabaoglu Y Hakimi AA Makarov V Pan-cancer analysis of intratumor heterogeneity as a prognostic determinant of survival. Oncotarget (2016) 7(9):10051–63. 10.18632/oncotarget.7067 26840267 PMC4891103

[54] Merlo LM Pepper JW Reid BJ Maley CC. Cancer as an evolutionary and ecological process. Nat Rev Cancer (2006) 6(12):924–35. 10.1038/nrc2013 17109012

[55] Tirosh I Izar B Prakadan SM Wadsworth MH II Treacy D Trombetta JJ Dissecting the multicellular ecosystem of metastatic melanoma by single-cell RNA-seq. Science (2016) 352(6282):189–96. 10.1126/science.aad0501 27124452 PMC4944528

[56] Marusyk A Tabassum DP Altrock PM Almendro V Michor F Polyak K. Non-cell-autonomous driving of tumour growth supports sub-clonal heterogeneity. Nature (2014) 514(7520):54–8. 10.1038/nature13556 25079331 PMC4184961

[57] Velazquez EF Jungbluth AA Yancovitz M Gnjatic S Adams S O’Neill D Expression of the cancer/testis antigen NY-ESO-1 in primary and metastatic malignant melanoma (MM) – correlation with prognostic factors. Cancer Immun (2007) 7:11. 17625806 PMC2935749

[58] Zhang J Fujimoto J Zhang J Wedge DC Song X Zhang J Intratumor heterogeneity in localized lung adenocarcinomas delineated by multiregion sequencing. Science (2014) 346(6206):256–9. 10.1126/science.1256930 25301631 PMC4354858

[59] Cogdill AP Andrews MC Wargo JA. Hallmarks of response to immune checkpoint blockade. Br J Cancer (2017) 117(1):1–7. 10.1038/bjc.2017.136 28524159 PMC5520201

[60] Dunn GP Bruce AT Ikeda H Old LJ Schreiber RD. Cancer immunoediting: from immunosurveillance to tumor escape. Nat Immunol (2002) 3(11):991–8. 10.1038/ni1102-991 12407406

[61] Swann JB Smyth MJ. Immune surveillance of tumors. J Clin Invest (2007) 117(5):1137–46. 10.1172/JCI31405 17476343 PMC1857231

[62] Spitzer MH Carmi Y Reticker-Flynn NE Kwek SS Madhireddy D Martins MM Systemic immunity is required for effective cancer immunotherapy. Cell (2017) 168(3):487–502.e15. 10.1016/j.cell.2016.12.022 28111070 PMC5312823

[63] Gabrilovich DI Nagaraj S. Myeloid-derived suppressor cells as regulators of the immune system. Nat Rev Immunol (2009) 9(3):162–74. 10.1038/nri2506 19197294 PMC2828349

[64] Kumar V Patel S Tcyganov E Gabrilovich DI The nature of myeloid-derived suppressor cells in the tumor microenvironment. Trends Immunol (2016) 37(3):208–20. 10.1016/j.it.2016.01.004 26858199 PMC4775398

[65] Florcken A Takvorian A Singh A Gerhardt A Ostendorf BN Dorken B Myeloid-derived suppressor cells in human peripheral blood: optimized quantification in healthy donors and patients with metastatic renal cell carcinoma. Immunol Lett (2015) 168(2):260–7. 10.1016/j.imlet.2015.10.001 26462434

[66] Zhang B Wang Z Wu L Zhang M Li W Ding J Circulating and tumor-infiltrating myeloid-derived suppressor cells in patients with colorectal carcinoma. PLoS One (2013) 8(2):e57114. 10.1371/journal.pone.0057114 23437326 PMC3577767

[67] Stanojevic I Miller K Kandolf-Sekulovic L Mijuskovic Z Zolotarevski L Jovic M A subpopulation that may correspond to granulocytic myeloid-derived suppressor cells reflects the clinical stage and progression of cutaneous melanoma. Int Immunol (2016) 28(2):87–97. 10.1093/intimm/dxv053 26391013 PMC4885218

[68] Wang L Chang EW Wong SC Ong SM Chong DQ Ling KL. Increased myeloid-derived suppressor cells in gastric cancer correlate with cancer stage and plasma S100A8/A9 proinflammatory proteins. J Immunol (2013) 190(2):794–804. 10.4049/jimmunol.1202088 23248262

[69] Gonda K Shibata M Ohtake T Matsumoto Y Tachibana K Abe N Myeloid-derived suppressor cells are increased and correlated with type 2 immune responses, malnutrition, inflammation, and poor prognosis in patients with breast cancer. Oncol Lett (2017) 14(2):1766–74. 10.3892/ol.2017.6305 28789407 PMC5529875

[70] Tanaka T Fujita M Hasegawa H Arimoto A Nishi M Fukuoka E Frequency of myeloid-derived suppressor cells in the peripheral blood reflects the status of tumor recurrence. Anticancer Res (2017) 37(7):3863–9. 10.21873/anticanres.11766 28668887

[71] Angell TE Lechner MG Smith AM Martin SE Groshen SG Maceri DR Circulating myeloid-derived suppressor cells predict differentiated thyroid cancer diagnosis and extent. Thyroid (2016) 26(3):381–9. 10.1089/thy.2015.0289 26756227 PMC4790214

[72] Raychaudhuri B Rayman P Ireland J Ko J Rini B Borden EC Myeloid-derived suppressor cell accumulation and function in patients with newly diagnosed glioblastoma. Neuro Oncol (2011) 13(6):591–9. 10.1093/neuonc/nor042 21636707 PMC3107102

[73] Vasquez-Dunddel D Pan F Zeng Q Gorbounov M Albesiano E Fu J STAT3 regulates arginase-I in myeloid-derived suppressor cells from cancer patients. J Clin Invest (2013) 123(4):1580–9. 10.1172/JCI60083 23454751 PMC3613901

[74] Porembka MR Mitchem JB Belt BA Hsieh CS Lee HM Herndon J Pancreatic adenocarcinoma induces bone marrow mobilization of myeloid-derived suppressor cells which promote primary tumor growth. Cancer Immunol Immunother (2012) 61(9):1373–85. 10.1007/s00262-011-1178-0 22215137 PMC3697836

[75] Brusa D Simone M Gontero P Spadi R Racca P Micari J Circulating immunosuppressive cells of prostate cancer patients before and after radical prostatectomy: profile comparison. Int J Urol (2013) 20(10):971–8. 10.1111/iju.12086 23421558

[76] Rodriguez PC Ernstoff MS Hernandez C Atkins M Zabaleta J Sierra R Arginase I-producing myeloid-derived suppressor cells in renal cell carcinoma are a subpopulation of activated granulocytes. Cancer Res (2009) 69(4):1553–60. 10.1158/0008-5472.CAN-08-1921 19201693 PMC2900845

[77] Gros A Parkhurst MR Tran E Pasetto A Robbins PF Ilyas S Prospective identification of neoantigen-specific lymphocytes in the peripheral blood of melanoma patients. Nat Med (2016) 22(4):433–8. 10.1038/nm.4051 26901407 PMC7446107

[78] Lee YK Mazmanian SK. Has the microbiota played a critical role in the evolution of the adaptive immune system? Science (2010) 330(6012):1768–73. 10.1126/science.1195568 21205662 PMC3159383

[79] Hooper LV Littman DR Macpherson AJ. Interactions between the microbiota and the immune system. Science (2012) 336(6086):1268–73. 10.1126/science.1223490 22674334 PMC4420145

[80] Backhed F Ley RE Sonnenburg JL Peterson DA Gordon JI. Host-bacterial mutualism in the human intestine. Science (2005) 307(5717):1915–20. 10.1126/science.1104816 15790844

[81] Gill SR Pop M Deboy RT Eckburg PB Turnbaugh PJ Samuel BS Metagenomic analysis of the human distal gut microbiome. Science (2006) 312(5778):1355–9. 10.1126/science.1124234 16741115 PMC3027896

[82] Kim D Zeng MY Nunez G. The interplay between host immune cells and gut microbiota in chronic inflammatory diseases. Exp Mol Med (2017) 49(5):e339. 10.1038/emm.2017.24 28546562 PMC5454439

[83] Neish AS. Microbes in gastrointestinal health and disease. Gastroenterology (2009) 136(1):65–80. 10.1053/j.gastro.2008.10.080 19026645 PMC2892787

[84] Marshall BJ Warren JR. Unidentified curved bacilli in the stomach of patients with gastritis and peptic ulceration. Lancet (1984) 1(8390):1311–5. 10.1016/S0140-6736(84)91816-6 6145023

[85] Wu S Rhee KJ Albesiano E Rabizadeh S Wu X Yen HR A human colonic commensal promotes colon tumorigenesis via activation of T helper type 17 T cell responses. Nat Med (2009) 15(9):1016–22. 10.1038/nm.2015 19701202 PMC3034219

[86] Arthur JC Perez-Chanona E Muhlbauer M Tomkovich S Uronis JM Fan TJ Intestinal inflammation targets cancer-inducing activity of the microbiota. Science (2012) 338(6103):120–3. 10.1126/science.1224820 22903521 PMC3645302

[87] Buc E Dubois D Sauvanet P Raisch J Delmas J Darfeuille-Michaud A High prevalence of mucosa-associated *E. coli* producing cyclomodulin and genotoxin in colon cancer. PLoS One (2013) 8(2):e56964. 10.1371/journal.pone.0056964 23457644 PMC3572998

[88] Kostic AD Gevers D Pedamallu CS Michaud M Duke F Earl AM Genomic analysis identifies association of *Fusobacterium* with colorectal carcinoma. Genome Res (2012) 22(2):292–8. 10.1101/gr.126573.111 22009990 PMC3266036

[89] Castellarin M Warren RL Freeman JD Dreolini L Krzywinski M Strauss J *Fusobacterium nucleatum* infection is prevalent in human colorectal carcinoma. Genome Res (2012) 22(2):299–306. 10.1101/gr.126516.111 22009989 PMC3266037

[90] McCoy AN Araujo-Perez F Azcarate-Peril A Yeh JJ Sandler RS Keku TO. *Fusobacterium* is associated with colorectal adenomas. PLoS One (2013) 8(1):e53653. 10.1371/journal.pone.0053653 23335968 PMC3546075

[91] Sanapareddy N Legge RM Jovov B McCoy A Burcal L Araujo-Perez F Increased rectal microbial richness is associated with the presence of colorectal adenomas in humans. ISME J (2012) 6(10):1858–68. 10.1038/ismej.2012.43 22622349 PMC3446812

[92] Bhatt AP Redinbo MR Bultman SJ. The role of the microbiome in cancer development and therapy. CA Cancer J Clin (2017) 67(4):326–44. 10.3322/caac.21398 28481406 PMC5530583

[93] Reddy BS Narisawa T Wright P Vukusich D Weisburger JH Wynder EL. Colon carcinogenesis with azoxymethane and dimethylhydrazine in germ-free rats. Cancer Res (1975) 35(2):287–90. 162868

[94] Vannucci L Stepankova R Kozakova H Fiserova A Rossmann P Tlaskalova-Hogenova H. Colorectal carcinogenesis in germ-free and conventionally reared rats: different intestinal environments affect the systemic immunity. Int J Oncol (2008) 32(3):609–17. 10.3892/ijo.32.3.609 18292938

[95] Grivennikov SI Wang K Mucida D Stewart CA Schnabl B Jauch D Adenoma-linked barrier defects and microbial products drive IL-23/IL-17-mediated tumour growth. Nature (2012) 491(7423):254–8. 10.1038/nature11465 23034650 PMC3601659

[96] Klimesova K Kverka M Zakostelska Z Hudcovic T Hrncir T Stepankova R Altered gut microbiota promotes colitis-associated cancer in IL-1 receptor-associated kinase M-deficient mice. Inflamm Bowel Dis (2013) 19(6):1266–77. 10.1097/MIB.0b013e318281330a 23567778 PMC3744230

[97] Dapito DH Mencin A Gwak GY Pradere JP Jang MK Mederacke I Promotion of hepatocellular carcinoma by the intestinal microbiota and TLR4. Cancer Cell (2012) 21(4):504–16. 10.1016/j.ccr.2012.02.007 22516259 PMC3332000

[98] Ochi A Nguyen AH Bedrosian AS Mushlin HM Zarbakhsh S Barilla R MyD88 inhibition amplifies dendritic cell capacity to promote pancreatic carcinogenesis via Th2 cells. J Exp Med (2012) 209(9):1671–87. 10.1084/jem.20111706 22908323 PMC3428946

[99] Iida N Dzutsev A Stewart CA Smith L Bouladoux N Weingarten RA Commensal bacteria control cancer response to therapy by modulating the tumor microenvironment. Science (2013) 342(6161):967–70. 10.1126/science.1240527 24264989 PMC6709532

[100] Viaud S Saccheri F Mignot G Yamazaki T Daillere R Hannani D The intestinal microbiota modulates the anticancer immune effects of cyclophosphamide. Science (2013) 342(6161):971–6. 10.1126/science.1240537 24264990 PMC4048947

[101] Vetizou M Pitt JM Daillere R Lepage P Waldschmitt N Flament C Anticancer immunotherapy by CTLA-4 blockade relies on the gut microbiota. Science (2015) 350(6264):1079–84. 10.1126/science.aad1329 26541610 PMC4721659

[102] Gopalakrishnan V Spencer CN Nezi L Reuben A Andrews MC Karpinets TV Gut microbiome modulates response to anti-PD-1 immunotherapy in melanoma patients. Science (2018) 359(6371):97–103. 10.1126/science.aan4236 29097493 PMC5827966

[103] Sivan A Corrales L Hubert N Williams JB Aquino-Michaels K Earley ZM Commensal *Bifidobacterium* promotes antitumor immunity and facilitates anti-PD-L1 efficacy. Science (2015) 350(6264):1084–9. 10.1126/science.aac4255 26541606 PMC4873287

[104] Daillere R Vetizou M Waldschmitt N Yamazaki T Isnard C Poirier-Colame V *Enterococcus hirae* and *Barnesiella intestinihominis* facilitate cyclophosphamide-induced therapeutic immunomodulatory effects. Immunity (2016) 45(4):931–43. 10.1016/j.immuni.2016.09.009 27717798

[105] Matson V Fessler J Bao R Chongsuwat T Zha Y Alegre ML The commensal microbiome is associated with anti-PD-1 efficacy in metastatic melanoma patients. Science (2018) 359(6371):104–8. 10.1126/science.aao3290 29302014 PMC6707353

[106] Frankel AE Coughlin LA Kim J Froehlich TW Xie Y Frenkel EP Metagenomic shotgun sequencing and unbiased metabolomic profiling identify specific human gut microbiota and metabolites associated with immune checkpoint therapy efficacy in melanoma patients. Neoplasia (2017) 19(10):848–55. 10.1016/j.neo.2017.08.004 28923537 PMC5602478

[107] Chaput N Lepage P Coutzac C Soularue E Le Roux K Monot C Baseline gut microbiota predicts clinical response and colitis in metastatic melanoma patients treated with ipilimumab. Ann Oncol (2017) 28(6):1368–79. 10.1093/annonc/mdx108 28368458

[108] Arpaia N Campbell C Fan X Dikiy S van der Veeken J deRoos P Metabolites produced by commensal bacteria promote peripheral regulatory T-cell generation. Nature (2013) 504(7480):451–5. 10.1038/nature12726 24226773 PMC3869884

[109] Furusawa Y Obata Y Fukuda S Endo TA Nakato G Takahashi D Commensal microbe-derived butyrate induces the differentiation of colonic regulatory T cells. Nature (2013) 504(7480):446–50. 10.1038/nature12721 24226770

[110] Usami M Kishimoto K Ohata A Miyoshi M Aoyama M Fueda Y Butyrate and trichostatin A attenuate nuclear factor kappaB activation and tumor necrosis factor alpha secretion and increase prostaglandin E2 secretion in human peripheral blood mononuclear cells. Nutr Res (2008) 28(5):321–8. 10.1016/j.nutres.2008.02.012 19083427

[111] Vinolo MA Rodrigues HG Hatanaka E Sato FT Sampaio SC Curi R. Suppressive effect of short-chain fatty acids on production of proinflammatory mediators by neutrophils. J Nutr Biochem (2011) 22(9):849–55. 10.1016/j.jnutbio.2010.07.009 21167700

[112] Smith PM Howitt MR Panikov N Michaud M Gallini CA Bohlooly YM The microbial metabolites, short-chain fatty acids, regulate colonic Treg cell homeostasis. Science (2013) 341(6145):569–73. 10.1126/science.1241165 23828891 PMC3807819

[113] Zitvogel L Ayyoub M Routy B Kroemer G. Microbiome and anticancer immunosurveillance. Cell (2016) 165(2):276–87. 10.1016/j.cell.2016.03.001 27058662

[114] Taur Y Jenq RR Perales MA Littmann ER Morjaria S Ling L The effects of intestinal tract bacterial diversity on mortality following allogeneic hematopoietic stem cell transplantation. Blood (2014) 124(7):1174–82. 10.1182/blood-2014-02-554725 24939656 PMC4133489

[115] Jenq RR Taur Y Devlin SM Ponce DM Goldberg JD Ahr KF Intestinal *Blautia* is associated with reduced death from graft-versus-host disease. Biol Blood Marrow Transplant (2015) 21(8):1373–83. 10.1016/j.bbmt.2015.04.016 25977230 PMC4516127

[116] Routy B Le Chatelier E Derosa L Duong CPM Alou MT Daillere R Gut microbiome influences efficacy of PD-1-based immunotherapy against epithelial tumors. Science (2018) 359(6371):91–7. 10.1126/science.aan3706 29097494

[117] Krishnan S Eslick GD. *Streptococcus bovis* infection and colorectal neoplasia: a meta-analysis. Colorectal Dis (2014) 16(9):672–80. 10.1111/codi.12662 24824513

[118] Mima K Nishihara R Qian ZR Cao Y Sukawa Y Nowak JA *Fusobacterium nucleatum* in colorectal carcinoma tissue and patient prognosis. Gut (2016) 65(12):1973–80. 10.1136/gutjnl-2015-310101 26311717 PMC4769120

[119] Bullman S Pedamallu CS Sicinska E Clancy TE Zhang X Cai D Analysis of *Fusobacterium* persistence and antibiotic response in colorectal cancer. Science (2017) 358(6369):1443–8. 10.1126/science.aal5240 29170280 PMC5823247

[120] Yu T Guo F Yu Y Sun T Ma D Han J *Fusobacterium nucleatum* promotes chemoresistance to colorectal cancer by modulating autophagy. Cell (2017) 170(3):548–63 e16. 10.1016/j.cell.2017.07.008 28753429 PMC5767127

[121] Eklof V Lofgren-Burstrom A Zingmark C Edin S Larsson P Karling P Cancer-associated fecal microbial markers in colorectal cancer detection. Int J Cancer (2017) 141(12):2528–36. 10.1002/ijc.31011 28833079 PMC5697688

[122] Vizcaino MI Crawford JM. The colibactin warhead crosslinks DNA. Nat Chem (2015) 7(5):411–7. 10.1038/nchem.2221 25901819 PMC4499846

[123] Tomkovich S Yang Y Winglee K Gauthier J Muhlbauer M Sun X Locoregional effects of microbiota in a preclinical model of colon carcinogenesis. Cancer Res (2017) 77(10):2620–32. 10.1158/0008-5472.CAN-16-3472 28416491 PMC5468752

[124] Lennard KS Goosen RW Blackburn JM. Bacterially-associated transcriptional remodelling in a distinct genomic subtype of colorectal cancer provides a plausible molecular basis for disease development. PLoS One (2016) 11(11):e0166282. 10.1371/journal.pone.0166282 27846243 PMC5112903

[125] Gur C Ibrahim Y Isaacson B Yamin R Abed J Gamliel M Binding of the Fap2 protein of *Fusobacterium nucleatum* to human inhibitory receptor TIGIT protects tumors from immune cell attack. Immunity (2015) 42(2):344–55. 10.1016/j.immuni.2015.01.010 25680274 PMC4361732

[126] Thiele Orberg E Fan H Tam AJ Dejea CM Destefano Shields CE Wu S The myeloid immune signature of enterotoxigenic *Bacteroides fragilis*-induced murine colon tumorigenesis. Mucosal Immunol (2017) 10(2):421–33. 10.1038/mi.2016.53 27301879 PMC5159334

[127] Geller LT Barzily-Rokni M Danino T Jonas OH Shental N Nejman D Potential role of intratumor bacteria in mediating tumor resistance to the chemotherapeutic drug gemcitabine. Science (2017) 357(6356):1156–60. 10.1126/science.aah5043 28912244 PMC5727343

[128] Wang H Altemus J Niazi F Green H Calhoun BC Sturgis C Breast tissue, oral and urinary microbiomes in breast cancer. Oncotarget (2017) 8(50):88122–38. 10.18632/oncotarget.21490 29152146 PMC5675698

[129] Rubinstein MR Wang X Liu W Hao Y Cai G Han YW *Fusobacterium nucleatum* promotes colorectal carcinogenesis by modulating E-cadherin/beta-catenin signaling via its FadA adhesin. Cell Host Microbe (2013) 14(2):195–206. 10.1016/j.chom.2013.07.012 23954158 PMC3770529

